# Identifying Less Burdensome and More Cost‐Efficient Incomplete Stepped Wedge Designs for Continuous Outcomes Collected via Repeated Cross‐Sections

**DOI:** 10.1002/sim.70067

**Published:** 2025-04-25

**Authors:** Ehsan Rezaei‐Darzi, Jessica Kasza, Anisa R. Assifi, Danielle Mazza, Andrew B. Forbes, Kelsey L. Grantham

**Affiliations:** ^1^ School of Public Health and Preventive Medicine Monash University Melbourne Australia; ^2^ SPHERE NHMRC Centre of Research Excellence, Department of General Practice, School of Public Health and Preventive Medicine Monash University Melbourne Australia

**Keywords:** cluster randomized trial, cost efficiency framework, optimization, repeated cross‐sectional, staircase design

## Abstract

Stepped wedge trials can be costly and burdensome. Recent work has investigated the iterative removal of cluster‐period cells from stepped wedge designs, producing a series of candidate incomplete designs that are less burdensome. We propose a novel way to explore the space of incomplete stepped wedge designs, by considering their cost efficiency, seeking to identify designs that retain high power while limiting the total trial cost. We define the cost efficiency of a design as the ratio of the precision of the treatment effect estimator to the total trial cost. Total trial cost incorporates the costs per cluster, costs per participant in intervention and control conditions, and the costs of restarting data collection in a cluster under intervention and control conditions following a pause. We consider linear mixed models for continuous outcomes with a repeated cross‐sectional sampling scheme and use an iterative procedure to remove individual cells with the lowest contribution to the cost efficiency metric, producing a series of progressively reduced designs. We define the optimal design within this design space as that which maximizes the cost efficiency relative to the complete design, subject to a minimum acceptable power constraint. We illustrate our methods with an example motivated by a real‐world trial. Our methods enable trialists to identify incomplete stepped wedge designs that are less burdensome and more cost‐efficient than complete designs. We find that “staircase”‐type designs, where clusters only contribute measurements immediately before and after the treatment switch, are often particularly cost‐efficient variants of the stepped wedge design.

## 1 Introduction

**FIGURE 1:**
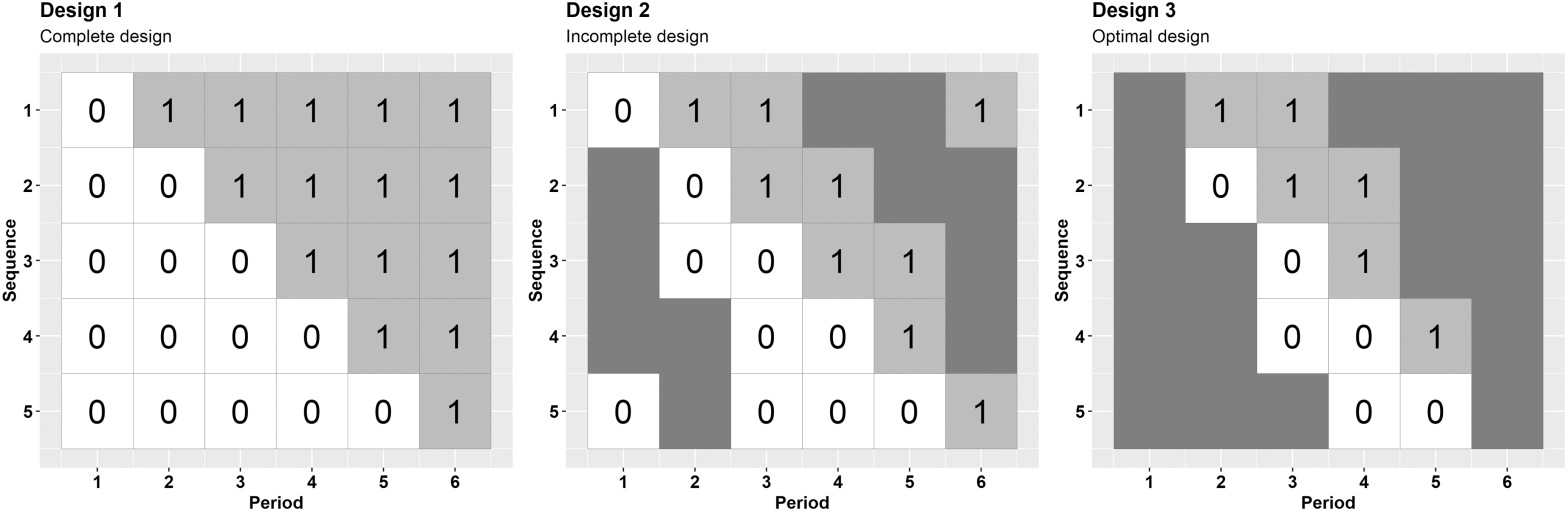
Schematic of selected designs for the ALLIANCE trial example with 5 sequences, where 8 pharmacies are assigned to the first and last sequences, and 7 pharmacies to the other sequences. Left: Design schematic of the complete stepped wedge design. Center: Design schematic of an incomplete design. Right: Design schematic of a staircase design, the optimal design. 0 indicates the control condition; 1 indicates the intervention condition.

The use of stepped wedge designs for evaluating interventions in public health and related disciplines is becoming increasingly common [1]. In the standard form of this design, clusters are randomized to different sequences; these sequences all start out by implementing the control condition and switching to the intervention condition in different periods of the trial until all sequences have implemented the intervention condition by the end of the trial, such as in Design 1 in Figure 1. However, with a standard stepped wedge design, all clusters are required to provide measurements for the entire duration of the trial: this may be costly and burdensome for clusters, and potentially also for individuals if they are followed over many trial periods, as occurs with a closed cohort sampling structure [2]. Fortunately, it may not be necessary for clusters to provide measurements in all periods of the trial: there is considerable variation in the amount of information that is contributed by each cluster in each period (i.e., by each “cluster‐period” of a stepped wedge design) to the estimation of the treatment effect [3]. Thus, efficient “incomplete” stepped wedge designs can be obtained from a complete design by removing those cluster‐period cells that contribute little information to the estimation of the treatment effect. An iterative procedure was recently developed that iteratively removes the cluster‐period cells that contribute the least information to the estimation of the treatment effect, yielding a series of progressively reduced incomplete designs [4]. Many of these designs provide nearly as much power as the complete design to detect effects of interest [4].

In addition to finding a design that yields adequate study power at the trial planning stage, it would be beneficial to consider the associated costs of various candidate designs: this leads to the planning of a trial that maximizes the statistical power to detect the treatment effect while minimizing total trial cost [5, 6]. Some methodology has been proposed to incorporate cost considerations into the planning of cluster randomized trials for various standard, or “complete” designs, such as the standard parallel cluster randomized trial design [7, 8, 9, 10, 11], the cluster randomized crossover (CRXO) design [6, 12, 13], or the stepped wedge design [13, 14]; we describe these approaches further below. In general, the cost of a cluster randomized trial will be influenced by a number of factors, including the number of clusters and the number of participants included in the trial [5]. Certain costs may also differ between the control and intervention conditions [10].

Several authors have incorporated trial cost considerations into the design of parallel or cluster crossover designs by recommending trialists select the most statistically efficient design among those with a total cost that does not exceed a given budget [6, 7, 9, 10, 11, 12, 15]. For parallel designs, where simple analytical expressions are available, a general strategy in finding optimal designs is to assume specific values for the correlation parameters; rearrange the trial cost formula in terms of one design characteristic; substitute into the precision expression; and then solve for a different trial characteristic [7, 10, 15, 16, 17, 18, 19]. In this setting, identifying the *optimal* design typically involves identifying both the number of clusters and cluster size that maximizes precision while considering any constraints on the total trial cost. An alternative method that allows for uncertainty in the correlation while incorporating cost considerations is to look for the “Maximin optimal design” [7, 8, 9, 10, 11]. This approach requires specification of a parameter space such as a range of plausible correlation values and a design space representing the trial configurations of interest. To find the Maximin optimal design over the parameter and design spaces, van Breukelen and Candel [7] outlined the following steps: for each correlation value in the parameter space, identify the design that has the highest precision given a budget constraint (the locally optimal design), and then calculate the relative efficiency of all designs in the design space in comparison to the locally optimal design. Then, for each design in the design space, calculate the minimum relative efficiency; and finally, across all designs in the design space, identify the design that gives the highest minimum relative efficiency value. Van Breukelen and Candel [7] used this approach to identify the Maximin optimal design for standard cluster randomized trials, the design with the optimal number of clusters and cluster size across a range of within‐cluster correlation values. For standard cluster randomized trials with binary outcomes, Wu et al. [8] derived the optimal proportion of clusters assigned to each treatment arm, where all clusters are of equal size, for a range of correlation values and success rates. In Wu et al. [8], the locally optimal design was the design that maximized a cost efficiency measure, defined as the ratio of the precision of the treatment effect estimator and the total study cost. For CRXO trials, Rietbergen and Moerbeek [12] took an analytical approach to find the optimal design given a fixed budget for a two‐period design when the number of clusters and cluster size can vary, assuming a block‐exchangeable correlation structure [20, 21]. They further discussed the minimal budget needed to achieve a specific power level with optimal allocation. Grantham et al. [6] numerically investigated the optimal number of crossovers for a fixed trial duration and number of participants per cluster when there is continuous recruitment and the similarity between participants' outcomes is assumed to continuously decay with greater distance between their measurement times. Grantham et al. [6] then assessed the efficiency of different designs relative to the optimal design, for designs with different numbers of clusters and periods that did not exceed a specified trial budget. Liu and Li [13] recently used a Maximin approach to find optimal multiple‐period CRXO trials under a cost efficiency framework when there is uncertainty in the correlation parameter values, applicable to closed cohort and repeated cross‐sectional designs.

Unlike parallel and CRXO designs, there has been minimal work to date incorporating costs into stepped wedge designs. Several papers have investigated the optimal allocation of clusters to sequences in the design of stepped wedge trials [20, 22, 23, 24, 25, 26], considering only design efficiency. However, Grayling et al. [14] considered the cost efficiency of multi‐arm stepped wedge designs by minimizing a weighted combination of total trial cost and a function of the covariance matrix of the treatment effect estimators. Liu and Li's algorithm discussed in the paragraph above was also used to explore optimal stepped wedge designs within a cost efficiency framework; however, their focus was on complete stepped wedge designs and they assumed a block‐exchangeable within‐cluster correlation structure [13], rather than any form of decaying correlation structure [27]. The cost efficiency of other types of stepped wedge designs, in particular incomplete designs, has not been investigated.

In this paper, we identify the most cost‐efficient incomplete stepped wedge design in the set of designs obtained by a greedy search maximizing a cost efficiency metric. Candidate incomplete designs are generated using an iterative approach guided by the cost efficiency metric, and the optimal design is the design within this set of incomplete designs that maximizes the cost efficiency relative to the complete stepped wedge design, and meets a minimum acceptable level of statistical power. These incomplete designs may include sequences that contain “gaps” made up of periods with no measurements. We provide a formula for total trial cost that incorporates the costs per cluster, per participant in intervention and control conditions, and a new cost relevant to incomplete designs, which is that of restarting data collection under intervention and control conditions in a cluster following a pause in data collection as dictated by any gaps in the design. Clusters may incur this “restart cost” when the personnel in the clusters need to undergo retraining in order to deliver an intervention, collect data, or record measurements after being inactive during the gaps.

The paper is organized as follows: in Section 2, we describe the statistical model for continuous outcomes, cost framework, the cost efficiency metric, the iterative removal of cells with low contributions to cost efficiency, and finding the optimal design within the design space. In Section 3, we provide examples of our approach utilizing cost values inspired by a real‐world stepped wedge trial, illustrate the example when there is uncertainty in correlation parameters, and we provide an online Shiny app to allow researchers to implement our approach in the context of their own trials. Our findings and conclusions are summarized in the final section.

## 2 Methods

### 2.1 Statistical Model

We consider stepped wedge designs that have repeated cross‐sectional sampling structures and constant cluster‐period sizes, with S unique sequences spanning a total of T=S+1 time periods and $N_{s}$ clusters randomly assigned to sequence s=1,…,S. We consider a continuous outcome and a linear mixed model with categorical period effects and a discrete‐time decay within‐cluster correlation structure. Under this correlation structure, the similarity between pairs of participants' outcomes within a cluster is assumed to decay as the distance in time between the participants' periods of measurement increases [27]. We suppose the measured outcome $Y_{\mathrm{kji}}$ for participant i=1,…,m measured in period j=1,…,T in cluster k=1,…,K (where $K=\sum_{s=1}^{S}N_{s}$) can be represented by the following model:

(1) $$\begin{array}{cc} & Y_{\mathrm{kji}}=\mu +\beta_{j}+X_{\mathrm{kj}}\theta +\gamma_{\mathrm{kj}}+\epsilon_{\mathrm{kji}}\\ & \epsilon_{\mathrm{kji}}∼N\left(0,\sigma_{\epsilon }^{2}\right),\;\gamma_{k}={\left(\gamma_{k1},\ldots ,\gamma_{\mathrm{kT}}\right)}^{T}∼N_{T}\left(0,\tau^{2}R\right) \end{array}$$

where μ is the overall mean outcome in the first period in the absence of treatment; $\beta_{j}$ is the fixed effect for period j with $\beta_{1}=0$ for identifiability; $X_{\mathrm{kj}}$ is the treatment indicator for cluster k in period j (0 for the control, 1 for the intervention); θ is the treatment effect; $\gamma_{k}={\left(\gamma_{k1},\ldots ,\gamma_{\mathrm{kT}}\right)}^{T}$ is the vector of cluster‐period random effects for cluster k; and $\epsilon_{\mathrm{kji}}$ is the participant‐level random error.

The covariance matrix for $\gamma_{k}$ is given by $\tau^{2}R$, where the $\left(j,j^{\prime }\right)$ element of R is given by $r^{\left|j-j^{\prime }\right|}$. This structure induces discrete‐time decay correlation, where the correlation between participants' outcomes measured in the same cluster but in different periods j and $j^{\prime }$ depends on the time between these periods: $\mathrm{cov}\left(\gamma_{\mathrm{kj}},\gamma_{kj^{\prime }}\right)=\tau^{2}r^{\left|j-j^{\prime }\right|}$ and so $\text{corr}\left(Y_{\mathrm{kji}},Y_{kj^{\prime }i^{\prime }}\right)=\frac{\tau^{2}}{\tau^{2}+\sigma_{\epsilon }^{2}}r^{\left|j-j^{\prime }\right|}=\rho r^{\left|j-j^{\prime }\right|}$, 0≤r≤1 [27]. The parameter ρ represents the correlation between two participants' outcomes measured within the same cluster in the same period and is referred to as the within‐period intracluster correlation (ICC), given by $\text{corr}\left(Y_{\mathrm{kji}},Y_{\mathrm{kj}i^{\prime }}\right)=\frac{\tau^{2}}{\tau^{2}+\sigma_{\epsilon }^{2}}$. The parameter r represents the proportionate reduction in correlation from one period to the next and is referred to as the cluster autocorrelation (CAC). The model with $r^{\left|j-j^{\prime }\right|}=r$ for all $j\neq j^{\prime }$ where $\text{corr}\left(Y_{\mathrm{kji}},Y_{kj^{\prime }i^{\prime }}\right)=\rho r$, represents the between‐period intracluster correlation, the correlation between two participants' outcomes within the same cluster measured in different periods [20, 21]. This correlation structure can be referred to as the “constant between‐period intracluster correlation” structure and has also been called a “nested‐exchangeable” correlation structure [28, 29]; we refer to this structure as the block‐exchangeable correlation structure [30]. When r=1, $\text{corr}\left(Y_{\mathrm{kji}},Y_{\mathrm{kj}i^{\prime }}\right)=\text{corr}\left(Y_{\mathrm{kji}},Y_{kj^{\prime }i^{\prime }}\right)=\rho$, and model (1) reduces to the exchangeable correlation model [31].

The variance of the estimator of the treatment effect, $\mathrm{var}\left(\hat{\theta }\right)$, is of interest at the trial design stage, as this quantity is needed to calculate the required sample size and statistical power. We make the typical choice of considering the estimator $\hat{\theta }$ that is obtained via generalized least squares. Let $T_{k}$ represent the number of measurement periods in which cluster k provides measurements, $Z_{k}$ symbolize the $T_{k}\times T$‐dimensional matrix encoding the parameterisation of the time effects corresponding to cluster k, taking the form of a T×T identity matrix with rows corresponding to unobserved periods removed, $V_{k}$ represent the $T_{k}\times T_{k}$ covariance matrix for cluster k and $X_{k}$ represent the $T_{k}\times 1$‐dimensional column vector of treatment indicators for the measurement periods for cluster k. Then the general expression that represents the variance of the treatment effect estimator, valid for both complete and incomplete stepped wedge designs [4] is represented as:

(2) $$\begin{array}{c} \mathrm{var}\left(\hat{\theta }\right)=\{\sum_{k=1}^{K}X_{k}^{T}V_{k}^{-1}X_{k}-{\left(\sum_{k=1}^{K}Z_{k}^{T}V_{k}^{-1}X_{k}\right)}^{T}\\ {{\left(\sum_{k=1}^{K}Z_{k}^{T}V_{k}^{-1}Z_{k}\right)}^{-1}\left(\sum_{k=1}^{K}Z_{k}^{T}V_{k}^{-1}X_{k}\right)\}}^{-1}\; \end{array}$$

Expression (2) illustrates how the covariance matrix and design parameters are combined to produce the variance of the treatment effect estimator. A more efficient computation is used in accompanying R code.

To test the null hypothesis $H_{0}:\theta =0$ versus the alternative hypothesis $H_{A}:\theta \neq 0$ assuming model (1), a Wald test using the test statistic $\hat{\theta }/\sqrt{\mathrm{var}\left(\hat{\theta }\right)}$, is commonly used. The asymptotic power [31], Pow, for conducting a two‐tailed test of size α to detect an effect size of $\theta_{d}$ is then given by:

$$\mathrm{Pow}=\Phi \left(\frac{\theta_{d}}{\sqrt{\mathrm{var}\left(\hat{\theta }\right)}}-z_{1-\frac{\alpha }{2}}\right)$$

where Φ represents the cumulative standard Normal distribution function and $z_{1-\frac{\alpha }{2}}$ is the $\left(1-\frac{\alpha }{2}\right)$ quantile of the standard Normal distribution function.

Alternatively, a *t*‐distribution could be used to calculate power and for the critical values, as may be appropriate when the number of clusters included in the study is small. However, in multiple‐period designs, the appropriate values for the degrees of freedom is unclear, as has been noted in Hemming et al. [32]. In that paper, the suggested degrees of freedom for multiple‐period designs was the number of cluster‐periods minus the number of time periods, minus one (i.e., KT−T−1). Furthermore, in incomplete stepped wedge designs, there is even less clarity in the appropriate choice for the degrees of freedom. Hence, we will not consider this approach further.

### 2.2 Cost Framework

In this section, we outline the costs relevant for an incomplete stepped wedge design and provide a formula for calculating its total trial cost. Incomplete stepped wedge designs may contain sequences with gaps of one or more periods of no measurement: clusters assigned to such a sequence would collect and provide data for one or more periods, pause data collection during the periods of the gap, and then resume data collection for one or more periods. This is illustrated in Design 2 in Figure 1: in sequence 1, data collection would temporarily stop during period 4 and resume in period 6 under the intervention condition, and in sequence 5, data collection would pause in periods 2 and resume in period 3 under the control condition. We suppose that clusters may incur some costs associated with resuming data collection following a pause, and so we include a restart cost in our total trial cost formula.

We consider the total cost of an incomplete stepped wedge design, where we account for the costs associated with the recruitment and measurement of individual participants, of entire clusters, of implementing the control and intervention conditions in clusters, and restarting data collection in a cluster following a gap in which no measurements are taken. We refer to participant‐related costs as the “cost per participant” which can include the costs associated with recruitment and data collection, and we allow this cost to differ between control and intervention periods. Additionally, the cost of restarting data collection may vary depending on whether the control or the intervention is being restarted, so we allow this to differ for the intervention and control conditions. We define the total trial cost for a design as a function of the cost per cluster (c), the costs of implementing the intervention and control conditions in a cluster (k, $k^{\prime }$, respectively), the costs per participant under the intervention and control conditions (p, $p^{\prime }$, respectively) and the cluster‐level restart costs under the intervention and control conditions (g, $g^{\prime }$, respectively). We consider a design consisting of a total of K clusters, with $N_{s}$ clusters allocated to sequence s, and with m participants measured in each cluster‐period of the design. Our focus is on incomplete designs where all clusters randomized to a sequence have the same pattern of control and intervention conditions, and so the following parameters depend only on s and will not vary across the clusters randomized to sequence s. We define $T_{s}$ and ${T^{\prime }}_{s}$ as the number of measurement periods observed in sequence s under the intervention and control conditions, respectively, $I_{s}$ as the indicator function for the presence of sequence s (since not all sequences may be present in a design), $I_{\mathrm{sI}}$ as an indicator for whether sequence s includes an intervention condition period, $I_{\mathrm{sC}}$ is the indicator for whether sequence s includes a control condition period, $n_{s}$ and ${n^{\prime }}_{s}$ as the number of gaps between measurement periods in sequence s, under the intervention and control conditions, respectively, noting that each stretch of one or more periods of no measurement with at least one period of measurement at either end is counted as a separate gap. In cases where multiple consecutive periods of no measurement (breaks) occur within a sequence, each distinct stretch of no measurement, separated by periods of measurement, should be considered a separate gap, that is, for Figure 1 Design 2, $T_{1}=3$, ${T^{\prime }}_{1}=1$, $n_{1}=1$, ${n^{\prime }}_{1}=0$ for the first sequence, and $T_{2}=2$, ${T^{\prime }}_{2}=1$ $n_{2}=0$, ${n^{\prime }}_{2}=0$ for the second sequence. Trial configuration and cost parameters are summarized in Table 1.

**TABLE 1:** Trial configuration and cost parameters for the trial examples.

Parameter	Definition	Values for the ALLIANCE trial	Values for the 15‐period design in Section 3.3	Values for the 15‐period design in Section 3.4
Parameters fixed across the series of reduced designs				
m	Number of participants measured in each cluster‐period	7	50	50
p	Cost per participant in the intervention group	$140	$80	$140
$p^{\prime }$	Cost per participant in the control group	$80	$80	$80
c	Cost of including a cluster in the trial	$2500	$2500	$2500
g	Restart cost under the intervention condition	$230	$2500	$0
$g^{\prime }$	Restart cost under the control condition	$0	$0	$0
k	Cost of implementing the intervention condition in a cluster	$0	$0	$0
$k^{\prime }$	Cost of implementing the control condition in a cluster	$0	$0	$0
Parameters that vary across the series of reduced designs (initial values of each shown)				
S	Total number of sequences that contribute measurements in at least one period	Initial value: 5	Initial value: 14	Initial value: 14
$T_{s}$	Number of measurement periods in sequence s under the intervention condition	Initial value: change according to s	Initial value: change according to s	Initial value: change according to s
${T^{\prime }}_{s}$	Number of measurement periods in sequence s under the control condition	Initial value: change according to s	Initial value: change according to s	Initial value: change according to s
$N_{s}$	Number of clusters randomized to sequence s	Initial value: 8 for s=1,5; 7 for s=2,3,4	Initial value: 1 for all s	Initial value: 1 for all s
$n_{s}$	Number of gaps in data collection in sequence s under the intervention condition	Initial value: 0	Initial value: 0	Initial value: 0
${n^{\prime }}_{s}$	Number of gaps in data collection in sequence s under the control condition	Initial value: 0	Initial value: 0	Initial value: 0
$I_{s}$	Indicator function for the presence of sequence s	Initial value: 1	Initial value: 1	Initial value: 1
$I_{\mathrm{sI}}$	Indicator function for whether sequence s includes an intervention period	Initial value: 1	Initial value: 1	Initial value: 1
$I_{\mathrm{sC}}$	Indicator function for whether sequence s includes a control period	Initial value: 1	Initial value: 1	Initial value: 1

We now describe how each of these elements is combined in order to determine the total trial cost for each design. First, the total number of clusters that contribute measurements in at least one period is multiplied by the cost of recruiting each cluster, c. Second, the total cluster‐level cost of implementing the intervention and control conditions can be obtained by multiplying each of the costs of implementing the intervention and control condition in a cluster by the corresponding total number of clusters that contribute measurements in at least one period, given by $k\sum_{s=1}^{S}N_{s}I_{\mathrm{sI}}+k^{\prime }\sum_{s=1}^{S}N_{s}I_{\mathrm{sC}}$. Subsequently, the total cost of including participants under the intervention and control conditions can be obtained by multiplying each of the participant costs by the corresponding total number of participants who are included in the design, which can be represented as $mp\sum_{s=1}^{S}N_{s}T_{s}+{\mathrm{mp}}^{\prime }\sum_{s=1}^{S}N_{s}{T^{\prime }}_{s}$. Lastly, the total cost of restarting data collection following gaps in the design can be obtained by multiplying each of the restart costs under the intervention and control conditions by the total number of corresponding gaps, given by $g\sum_{s=1}^{S}N_{s}n_{s}+g^{\prime }\sum_{s=1}^{S}N_{s}{n^{\prime }}_{s}$. The total cost of a potentially incomplete design with S sequences and $N_{s}$ clusters randomly allocated to sequence s is thus given by

(3) $$\begin{array}{cc} C & =c\sum_{s=1}^{S}N_{s}I_{s}+k\sum_{s=1}^{S}N_{s}I_{\mathrm{sI}}+k^{\prime }\sum_{s=1}^{S}N_{s}I_{\mathrm{sC}}+\mathrm{mp}\sum_{s=1}^{S}N_{s}T_{s}\\ & \;+{\mathrm{mp}}^{\prime }\sum_{s=1}^{S}N_{s}{T^{\prime }}_{s}+g\sum_{s=1}^{S}N_{s}n_{s}+g^{\prime }\sum_{s=1}^{S}N_{s}{n^{\prime }}_{s} \end{array}$$

### 2.3 The Cost Efficiency Metric

We define the cost efficiency of a given design as in Wu et al. [8]. That is, the cost efficiency of a given design is defined as the ratio of the precision of the treatment effect estimator, that is, the reciprocal of the variance of the treatment effect estimator, to the total trial cost. Letting C(D) denote the total trial cost and ${\mathrm{var}}_{D}\left(\hat{\theta }\right)$ denote the variance of the treatment effect estimator for design D, we define the cost efficiency of design D as:

(4) $$\mathrm{CE}\left(D\right)=\left(1/{\mathrm{var}}_{D}\left(\hat{\theta }\right)\right)/C\left(D\right)=\frac{1}{{\mathrm{var}}_{D}\left(\hat{\theta }\right)}\times \frac{1}{C\left(D\right)}$$

The higher CE(D), the better: larger values are associated with more cost‐efficient designs.

### 2.4 Iterative Removal of Cells With Low Contributions to Cost Efficiency: Defining the Design Space

For a given complete stepped wedge design, we now seek to find an incomplete version that has high cost efficiency as defined by Equation (4). For any complete stepped wedge design, there are a potentially very large number of incomplete variants of this design: for a stepped wedge design with T periods and S sequences, there are $2^{S\times T}$ potential incomplete designs (we note that not all of these incomplete designs will be valid, in the sense that it can provide an estimate of the treatment effect). Thus we seek to reduce the number of incomplete stepped wedge designs considered by defining an algorithm to move through the set of incomplete designs. The algorithm we define is a greedy search of the space, iteratively producing designs that maximize the cost efficiency metric given in Equation (4). Let 𝒟 denote the design space, that is, the sequence of the incomplete designs obtained by the algorithm. To move through the space of all incomplete stepped wedge designs, we iteratively remove sequence‐period cells, rather than cluster‐period cells, from the complete stepped wedge design until the resulting design does not permit estimation of the treatment effect. We consider the situation where $N_{s}$ clusters are randomized to sequence s of the standard stepped wedge design, and we remove entire sequence‐periods from the design, where each sequence‐period includes $N_{s}$ cluster‐periods.

The iterative removal procedure, where one sequence‐period cell is removed at each iteration, proceeds as follows. Start with a complete stepped wedge design, which we denote by $D_{0}$. For each sequence‐period cell a, denote the design with cell a removed as $D_{0}\left[a\right]$. Then calculate the cost efficiency of design $D_{0}\left[a\right]$ which we denote as $\mathrm{CE}\left(D_{0}\left[a\right]\right)$. We then select the sequence‐period cell $a^{*}$ that maximizes this metric; that is, the cell whose removal leads to the reduced design with the highest cost efficiency, and this defines the next design in the iterative procedure, $D_{1}=D_{0}\left[a^{*}\right]$. That is, we remove the cell that contributes the least in terms of the cost efficiency metric: this could be a particularly expensive cell that contributes little information to the estimation of the treatment effect. This process is repeated iteratively. At each step: for each remaining sequence‐period cell we again calculate the cost efficiency of the design with that cell removed, remove the cell that yields the highest cost efficiency, and continue until there is a design in which the treatment effect is no longer estimable. The steps of this algorithm are as follows:

1. Initial design reduction:
  - For each sequence‐period cell a in the complete design $D_{0}$, calculate the cost efficiency of the initial design $D_{0}$ with that sequence‐period cell a removed: $\mathrm{CE}\left(D_{0}\left[a\right]\right)$.
  - Identify the sequence‐period cell $a^{*}$ that leads to the reduced design with the highest cost efficiency.
  - Select the reduced design $D_{1}=D_{0}\left[a^{*}\right]$.
2. Subsequent reduced designs: Iterate l=2,… up to T(T−1)−1=L:
  - For each sequence‐period cell a in the design $D_{l-1}$, calculate the cost efficiency metric for each design with that cell removed: $\mathrm{CE}\left(D_{l-1}\left[a\right]\right)$.
  - Identify the sequence‐period cell $a^{*}$ that leads to the reduced design with the highest cost efficiency.
  - Select the reduced design $D_{l}=D_{l-1}\left[a^{*}\right]$.

The set of reduced designs obtained from these steps forms the design space $𝒟:\left\{D_{1},D_{2},\ldots ,D_{L}\right\}$. The procedure ends when the minimally viable design, that is, the design with the fewest measured cells that allows for estimation of the treatment effect, is obtained. Note that with varying participant and/or restart costs between intervention and control conditions, the total number of participants and/or gaps in data collection may not be equal under the intervention and control conditions.

### 2.5 Finding the Optimal Design Within Design Space 𝒟

For any particular design configuration, we aim to find the incomplete design within the design space 𝒟 that offers the highest cost efficiency compared to the complete design, while also ensuring the design has adequate power. To compare the cost efficiency of all of the designs $D_{l}$ in the series to that of the complete design $D_{0}$, we define the relative cost efficiency (RCE) of the designs as

(5) $$\mathrm{RCE}\left(D_{0},D_{l}\right)=\mathrm{CE}\left(D_{l}\right)/\mathrm{CE}\left(D_{0}\right)=\frac{C\left(D_{0}\right)}{C\left(D_{l}\right)}/\frac{{\mathrm{var}}_{D_{l}}\left(\hat{\theta }\right)}{{\mathrm{var}}_{D_{0}}\left(\hat{\theta }\right)}$$

As the ratio of two positive quantities (a ratio of costs and a ratio of variances), $\mathrm{RCE}\;\left(D_{0},D_{l}\;\right)\geq 0$. Given that results in Kasza and Forbes [3] indicate that for the linear mixed models that we consider, the variance of an incomplete stepped wedge design is always greater than the variance of the complete stepped wedge design, the denominator of the RCE (the variance ratio) is always ≥ 1. The cost ratio is limited below by 0. In the absence of restart costs (i.e., when $g_{s}=g_{s}^{\prime }=0$), the cost of an incomplete design will always be less than the cost of a complete design, and in this case, the numerator of the RCE will be ≥ 1. The RCE ratio is bounded above by the cost ratio between the complete design and the minimally viable design, resulting in the highest cost ratio, and assuming the lowest possible value for the variance ratio of 1. The aim is to find an incomplete design with a variance that is close to that of the complete design, while keeping total trial cost low. Thus, the goal is to maximize the RCE.

By assessing how the total cost of a trial changes in conjunction with design efficiency, trialists can identify cost‐efficient incomplete designs that may be preferable to a complete stepped wedge design. We will denote the power of design $D_{l}$ by $\mathrm{Pow}\left(D_{l}\right)$, and will suppose that there is a minimum level of acceptable statistical power, ${\mathrm{Pow}}_{\min}$, that the preferred design must meet to be of interest (which may often be 80%). Our optimization problem is then to find the design $D^{*}$ within design space 𝒟 that maximizes the RCE while adhering to the power constraint:

(6) $$D^{*}={\text{argmax}}_{\left\{D_{l}\in 𝒟:\mathrm{Pow}\;\left(D_{l}\right)\geq {\mathrm{Pow}}_{\min}\right\}}\left(\mathrm{RCE}\left(D_{0},D_{l}\right)\right)$$

We illustrate finding the optimal design $D^{*}$ in the next section.

## 3 Illustrative Examples

### 3.1 The ALLIANCE Trial Example

We consider an example inspired by the ALLIANCE stepped wedge trial [33]. The ALLIANCE trial assesses whether provision of contraceptive counseling within pharmacies increases the use of hormonal or intrauterine contraception among women seeking medical abortion or emergency contraception, compared to the standard dispensing of medication. The ALLIANCE trial randomized 37 pharmacies to a 5‐sequence stepped wedge design, with 8 pharmacies allocated to the first and last sequences and 7 pharmacies to the other sequences, all including transition periods. We maintain the same allocation of pharmacies to each sequence but exclude transition periods. Figure 1 (left) depicts the design schematic. As in the ALLIANCE trial, we assume that 7 participants are included in each pharmacy during each period of the trial.

In our example, we utilize cost values (in Australian dollars) inspired by the ALLIANCE trial, provided in Table 1. The cost per cluster, c=$2500, includes payments to the participating pharmacist and pharmacy, and the costs of training pharmacists in the delivery of the contraceptive counseling sessions. The cost per participant in the intervention condition is p=$140 and in the control condition is $p^{\prime }=\text{\$}80$. These differential participant costs are due to the additional cost of providing contraceptive counseling to the participants recruited under the intervention condition. There are no additional cluster‐level costs to implement the intervention or control conditions, so $k=k^{\prime }=0$. The actual ALLIANCE trial was planned as a standard stepped wedge design, and as such, costs of restarting data collection in a cluster after a pause were not considered by the ALLIANCE investigators. We assume that the restart cost under the intervention condition is g=$230, representing the cost of providing one training session to a pharmacist to refresh their knowledge of the provision of contraceptive counseling sessions after a break in their delivery. We assume that there is no cost to restarting data collection under the control condition, with $g^{\prime }=0$.

We consider model (1) for a continuous outcome, assuming a discrete‐time decay intracluster correlation structure, with an estimated within‐period ICC of ρ=0.05 and CAC of r=0.95, corresponding to a 5% decay in correlation from one period of measurement to the next. We specify a standardized effect size of 0.26, which yields power for the complete stepped wedge design of approximately 90% when considering a two‐sided significance level of 0.05. The total trial cost for the complete stepped wedge design is $263440.

**FIGURE 2:**
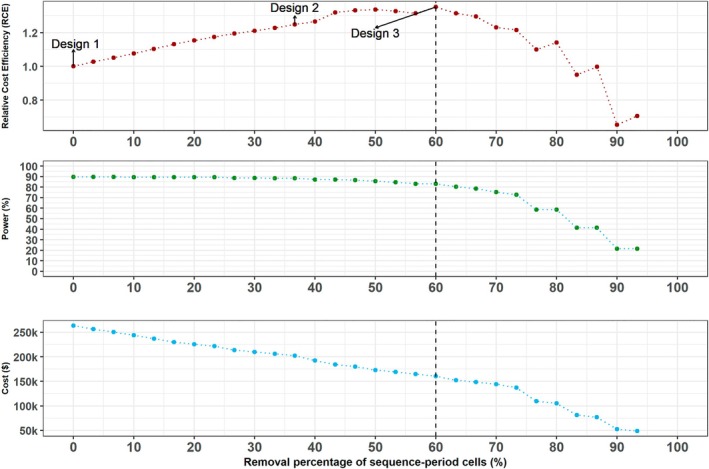
Relative cost efficiency (top panel), power to detect an effect size of 0.26 (middle panel), and total cost (bottom panel) corresponding to designs obtained through the iterative removal procedure for the ALLIANCE trial example. The design with 60% of the sequence‐period cells removed has the highest RCE and has power of at least 80%.

We searched the space of incomplete designs as described in Section 2.4 to generate a series of incomplete designs; we then obtained the optimal design as in Section 2.5 for a minimum acceptable power of 80%. Design 1 in Figure 1 displays the complete design, Design 2 in Figure 1 displays an incomplete design illustrating gaps in data collection, and Design 3 in Figure 1 shows the optimal design which has the highest RCE value and sufficient power. In Figure 2 (top) we display the RCE for all the designs in the series, and Figure 2 (middle and bottom) displays the corresponding power and the total trial cost. The optimal design has a RCE of 1.35 and retains around 40% of the sequence‐period cells of the complete design with no gaps in data collection. This design has power of 83% and reduces trial costs by almost 40%: from $263440 to $160260. This preferred design resembles a staircase design, an incomplete design where clusters contribute measurements solely around the main diagonal of the complete stepped wedge [34].

### 3.2 Selecting a Design When There Is Uncertainty in Correlation Parameters

**FIGURE 3:**
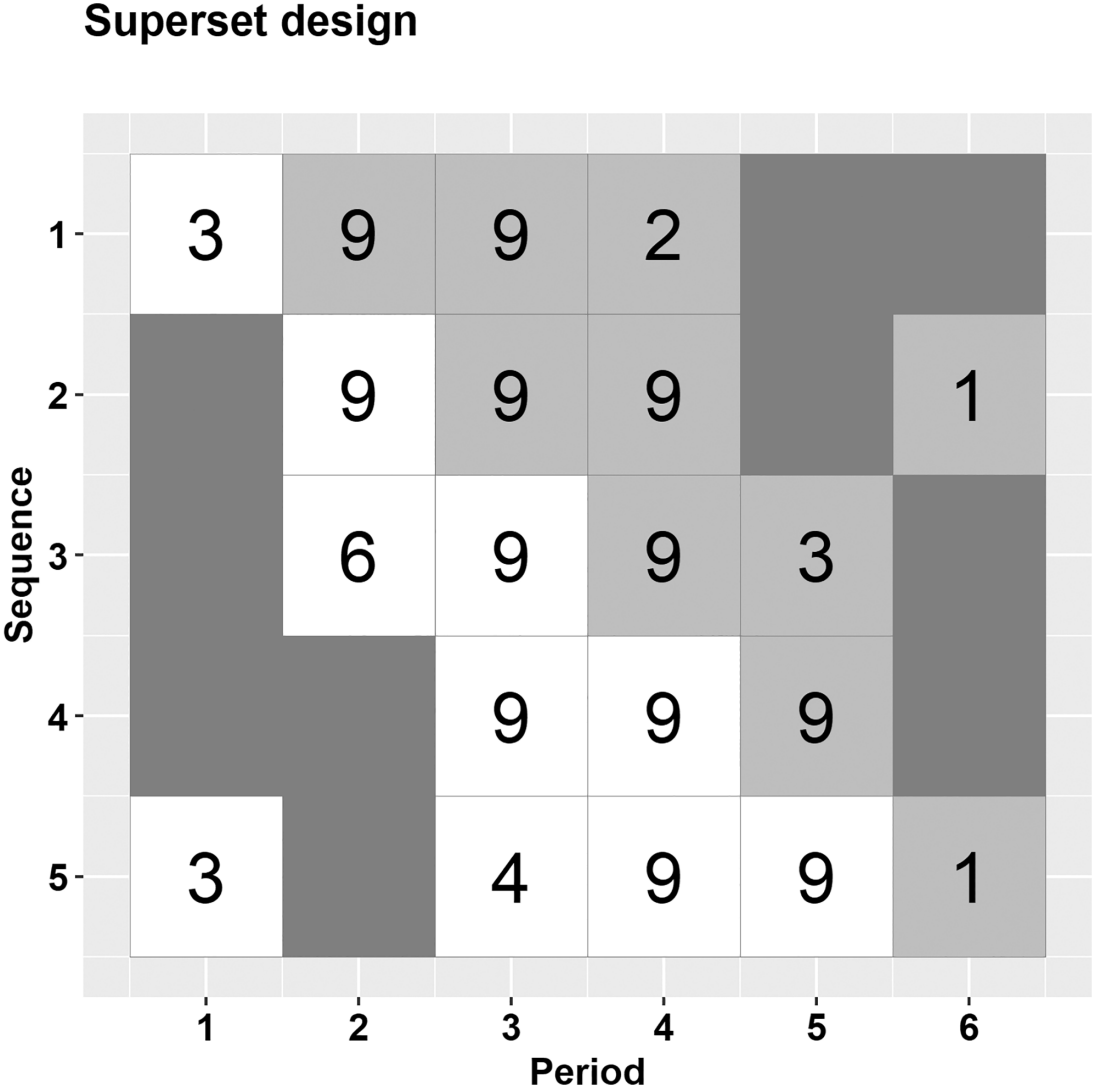
Schematic of the superset design for the ALLIANCE trial example. The numbers inside each sequence‐period cell indicate the number of times that each cell appeared in the set of optimal designs for the 9 combinations of ICC (0.01,0.05,0.1) and CAC (0.8,0.9,0.95). White color indicates the control condition; light gray color indicates the intervention condition.

In many cases, researchers face uncertainty regarding the values of correlation parameters, and thus, when selecting a design, they often evaluate designs for a range of potential choices for these parameters. To handle such situations, we have developed a strategy for the application of our iterative procedure to guide the selection of a cost‐efficient design. We provide an illustration of how our algorithm can be pragmatically applied in such scenarios. Our pragmatic approach involves first selecting a set of different correlation parameters for consideration, then applying the iterative procedure for each combination of this set of correlation parameters. To determine a set of plausible values for correlation parameters, the CLustered OUtcome Dataset (CLOUD) bank repository can serve as a useful resource [35]. The companion online tool reports correlation value estimates obtained from fitting linear mixed models with different correlation structures to real cluster randomized trial datasets. In this way, an optimal design corresponding to each element of this set of correlation parameters will be identified: that is, a set of optimal designs will be identified. We then propose a final “superset” design that includes all of the non‐missing sequence‐period cells from this set of optimal designs. This selected design will have the minimum acceptable level of power across all of the considered correlation choices and will have greater cost efficiency than the complete design. We now apply the following steps to the ALLIANCE trial:

1. Define the set of considered correlations: For the ALLIANCE trial, we consider the ICC to range between 0.01 and 0.1, with selected plausible values being 0.01, 0.05, 0.1 and the CAC to range between 0.8 and 0.95, with selected values of 0.95, 0.9, and 0.8. These choices were guided by the CLOUD bank repository [35]. For each of these nine parameter combinations (i.e., each combination of ICC and CAC) within the parameter space, we calculated the initial study power. Here, the lowest initial power was 82.8% for an ICC of 0.1 and a CAC of 0.8, while the highest was 94.7% for an ICC of 0.01 and a CAC of 0.8.
2. Find optimal designs: For each combination of ICC and CAC values defined in step (1) and for a minimum acceptable power of 80%, we then identified the optimal design within the design space 𝒟 as described in Section 2.5.
3. Combine the optimal designs: We then unified the contributing measurements from the set of optimal designs identified in Step (2). The design with all those contributing measurements, along with the number of times each sequence‐period cell was included in the optimal design is shown in Figure 3. In this example if we consider the design that incorporates only those cells that were included for all 9 combinations of ICC and CAC, then the final superset design is a variant of a staircase design.

Since each optimal design from all possible combinations (3×3) achieves at least 80% power, the superset design ensures that the power will be maintained at a minimum of 80%. The superset design achieves its highest study power of 93.6% with an ICC of 0.01 and its lowest study power of 81.2% with an ICC of 0.1, both with a CAC of 0.8. The total trial cost for this design is $208630 (recall that the cost of the complete design was $263440). The superset design in this example shows that cells around the treatment switch frequently appear in the optimal designs for the selected combinations of ICC and CAC values within the parameter space. We observe that, rather than unifying the contributing measurements from the optimal designs for all 9 combinations, this example only requires unifying those from the designs with ICC values of 0.01 and 0.1 and a CAC of 0.8 in creating the superset design. The optimal designs for each pair of ICC and CAC are provided in Section S1 of Data S2.

In certain trial settings, trialists may find in step 2 that some complete stepped wedge designs do not yield adequate power (i.e., the power of the complete design is below ${\mathrm{Pow}}_{\min}$) in which case none of the incomplete designs derived from that design will have adequate power either. Trialists may need to modify the trial configuration for the complete design, if feasible, and then follow the steps again to identify an appropriate superset design. If adjustments to the trial configuration are not possible, a complete design may need to be used.

### 3.3 Optimal Design for a Larger Complete Stepped Wedge Design

We also illustrate our proposed method for a larger initial stepped wedge design with 14 sequences (including one cluster per sequence) spanning 15 periods, with 50 participants measured in each cluster‐period. We again assume a discrete‐time decay within‐cluster correlation structure, but now with a large within‐period ICC of 0.15 and a CAC of 0.8. To illustrate a scenario where the cost of recommencing data collection after a pause would be particularly expensive, for example, as expensive as recruiting a new cluster, we opt for a restart cost of $2500 under the intervention condition. Such a situation could occur if recommencing data collection required extensive training of new staff, for example. Additionally, we assume equal costs of $80 per participant under both intervention and control conditions, and a restart cost of $0 under the control condition to evaluate the impact of having a nonzero restart cost solely under the intervention condition. The total cost of the complete stepped wedge design here is $875000.

**FIGURE 4:**
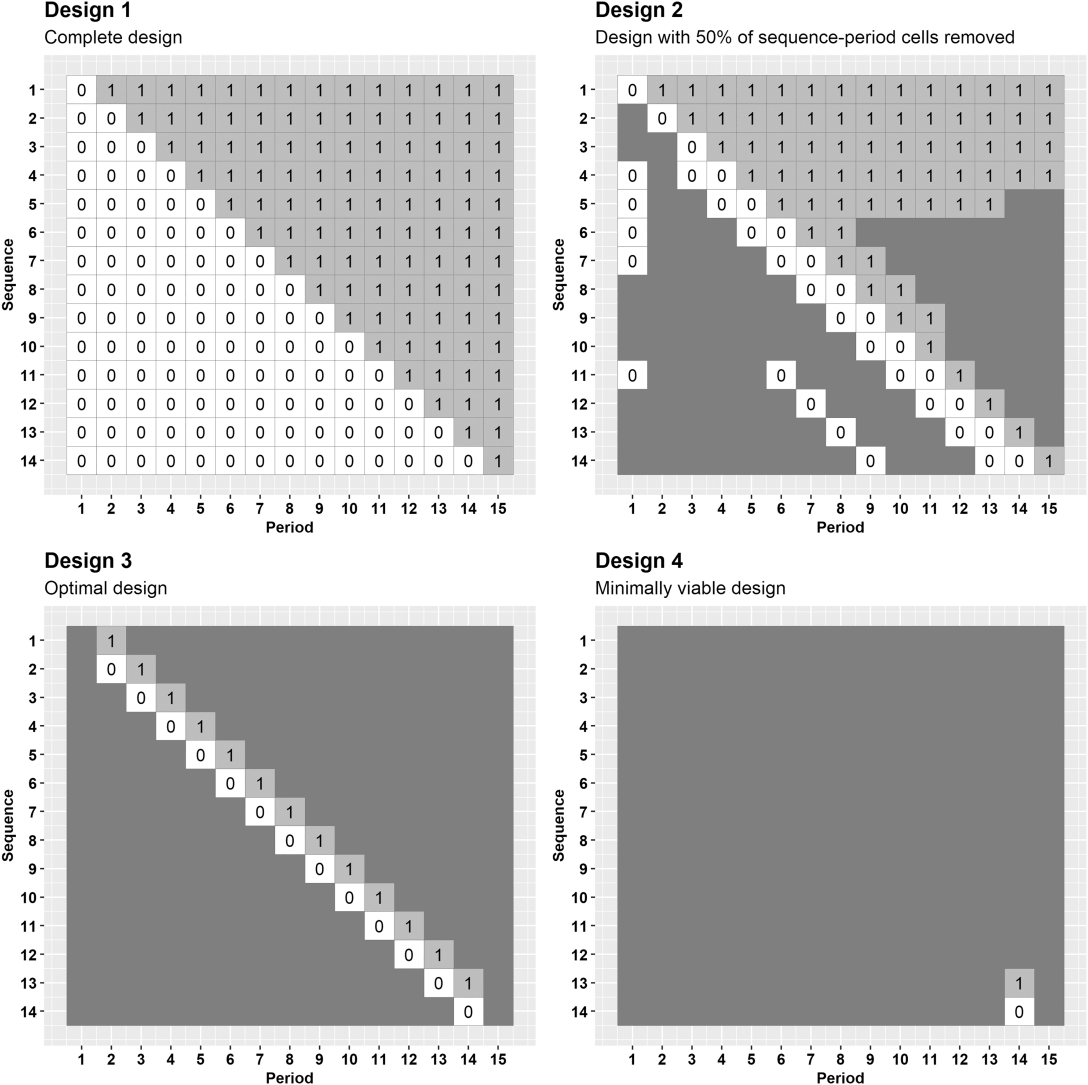
Select designs obtained by applying the iterative removal algorithm to the large trial example in Section 3.3. Top‐left: Design schematic of Design 1, a 14‐sequence (one cluster per sequence), 15‐period complete stepped wedge design. Top‐right: Design schematic of Design 2, the incomplete stepped wedge design obtained when 50% of sequence‐period cells from Design 1 have been removed. Bottom‐left: Design schematic of Design 3, the optimal design with the highest RCE and at least 80% power (87.6% sequence‐period cells removed). Bottom‐right: Design schematic of Design 4, the minimally viable design.

**FIGURE 5:**
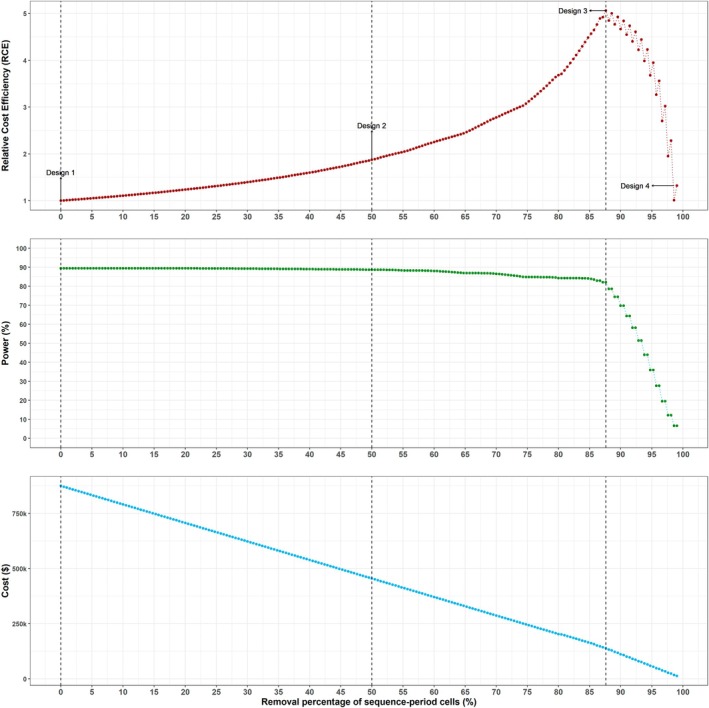
Relative cost efficiency (top panel), power to detect an effect size of 0.26 (middle panel), and total cost (bottom panel) corresponding to the series of designs obtained through applying the iterative removal procedure to the larger trial example in Section 3.3. The design with 87.6% of the sequence‐period cells removed has the highest RCE and has power of at least 80%.

For this design configuration and costs, we obtain the series of cost‐efficient incomplete designs comprising the design space 𝒟 following the approach in Section 2.4, and for a standardized effect size of 0.26, we identify the design that maximizes the RCE subject to a minimum acceptable power of 80% to find the optimal design. Figure 4 displays the design schematics for four select designs from this design space 𝒟 and Figure 5 displays the RCE, power and cost of each design, identified by removal percentage of sequence‐period cells. The complete design (Figure 4, Design 1) has 89.5% power to detect the effect size of 0.26. Design 2 in Figure 4, which retains 50% of the sequence‐period cells and includes a total of 9 gaps, has an RCE of 1.9 (as shown in Figure 5). The optimal design within the design space 𝒟 is depicted in Design 3 of Figure 4, with an RCE of 5.1 and 82.1% power. This design retains less than 13% of sequence‐period cells with no gaps in data collection and costs 84% less than the complete design ($139000). Once again, this optimal design resembles a staircase design [34].

### 3.4 Further Designs and Considerations

**FIGURE 6:**
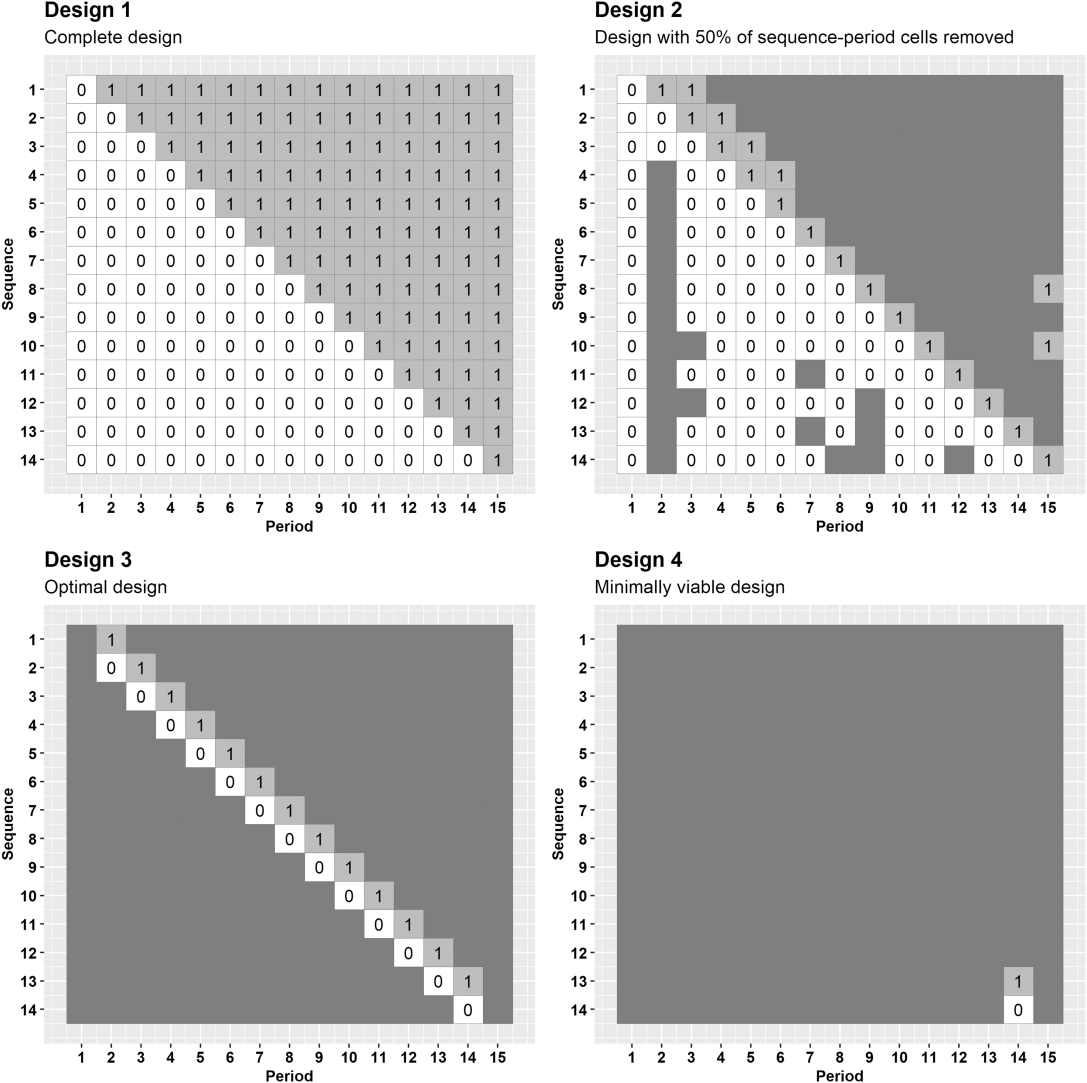
Select designs obtained by applying the iterative removal algorithm to the trial example with no restart cost under both conditions in Section 3.4. Top‐left: Design schematic of Design 1, a 14‐sequence (one cluster per sequence), 15‐period complete stepped wedge design. Top‐right: Design schematic of Design 2, the incomplete stepped wedge design obtained when 50% of sequence‐period cells from Design 1 have been removed. Bottom‐left: Design schematic of Design 3, the optimal design with the highest RCE and at least 80% power (87.6% of sequence‐period cells removed). Bottom‐right: Design schematic of Design 4, the minimally viable design.

**FIGURE 7:**
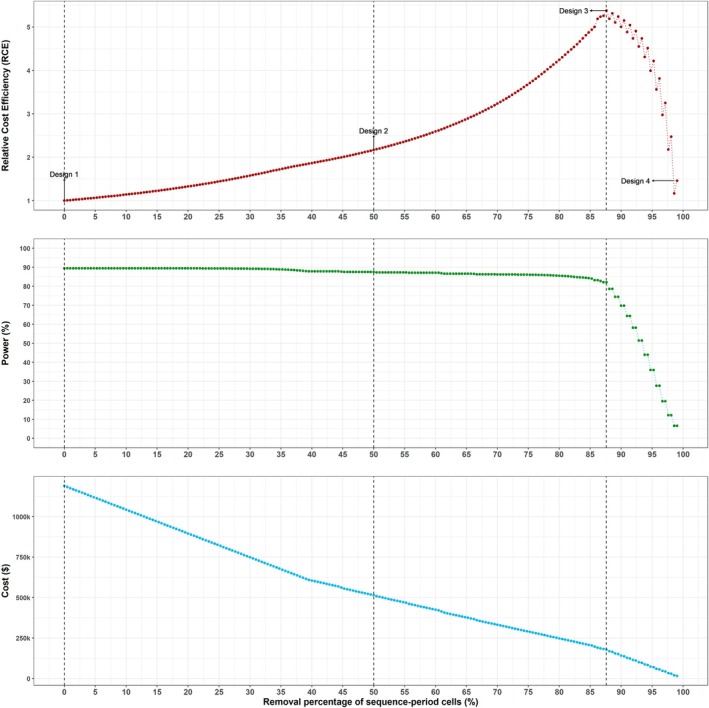
Relative cost efficiency (top panel), power to detect an effect size of 0.26 (middle panel), and total cost (bottom panel) corresponding to the series of designs obtained through applying the iterative removal procedure to the trial example with no restart cost under both conditions in Section 3.4. Design 3 is the optimal design, with 87.6% of the sequence‐period cells removed, the highest RCE and power of at least 80%.

To gain deeper insights into the pattern of generating incomplete designs and progressively removing low‐benefit sequence‐period cells, we now also illustrate a situation in which the cost of restarting data collection is $0 under both conditions. This example provides an illustration of the fact that in the absence of restart costs, incomplete designs that include sequences with gaps are more likely to emerge throughout the removal process. We consider the same complete stepped wedge design and the same correlation parameters as in Section 3.3, for the same values of the remaining cost parameters as in Section 3.1, again for a standardized effect size of 0.26 and minimum acceptable power of 80%. Design 1 in Figure 6 shows the complete design and Design 2 has 50% of the sequence‐period cells remaining, includes a total of 19 gaps, and has an RCE of 2.2 (see Figure 7). The zero cost of restarting data collection enables this design to have gaps in data collection without increasing trial costs. The optimal design is shown in Design 3 and resembles a staircase design, with the highest RCE of 5.4 in the series and 82.1% power to detect the treatment effect.

When there are costs associated with restarting data collection, the algorithm avoids designs with gaps. In other words, the restart cost impacts the number and arrangement of gaps. With a low restart cost, gaps can occur between the measurement periods for each sequence. Conversely, a high restart cost leads to an incomplete design with fewer gaps by focusing on removing cells from the sequence's boundaries.

### 3.5 A Shiny App to Identity the Most Cost‐Efficient Design

While only a limited set of trial configurations have been explored in this paper, we also provide an online app (https:// monash‐biostat.shinyapps.io/Iterative‐removals‐CE‐diffcl/) to allow readers to find the most cost‐efficient design subject to a given power constraint within the design space 𝒟 for a range of design configuration and cost parameters. Users can specify the number of periods, number of participants in each cluster in each period, number of clusters allocated to each sequence, which may differ across sequences, the type of correlation structure, within‐period intracluster correlation ICC, cluster autocorrelation CAC, and the effect size of interest. Additionally, users can specify the cost per cluster, costs per participant recruited under each of the control and intervention conditions, and the restart costs under each of the control and intervention conditions. We allow users to specify the minimum acceptable power level to determine the optimal design. Users can choose to view only those designs that exceed this minimum acceptable power by ticking a checkbox or if they would prefer to obtain the full series of incomplete designs, they can leave it unchecked. Users can view the designs in the series by iteration number, the optimal design selected by the algorithm, as well as plots of RCE, power, variance, cost, and precision loss.

## 4 Discussion

In this paper we have described a novel way to explore the space of incomplete stepped wedge designs, defined by iteratively removing sequence‐period cells from the complete design whose removal leads to a design with the highest cost efficiency. Earlier work focusing on design precision found that incomplete stepped wedge designs with as few as half of the cluster‐period cells from the complete design can be highly efficient, with only small reductions in precision [4]. These designs tend to have measurements concentrated around the time of the treatment switch (and possibly in the corners of the design) and may include pauses in data collection in some sequences. By incorporating costs into our algorithm for the selection of an incomplete design, our current work allows statisticians and trialists who are planning these trials to develop a more complete understanding of incomplete stepped wedge designs that are appropriate for their setting. We have found in this paper that incomplete designs resembling staircase designs, where clusters only contribute measurements immediately before and after the treatment switch, are particularly cost‐efficient designs relative to the complete stepped wedge designs, often retaining high levels of study power for a substantially lower total trial cost.

Although the examples that we considered in Section 3 indicate that staircase designs are cost‐efficient and can provide sufficient power and high precision, such designs are not always the optimal choice. While a discrete‐time decay correlation structure was considered for the examples in Sections 3.3 and 3.4 in this paper, if an exchangeable correlation structure were assumed instead, optimal designs resembled staircase designs together with measurements in the corners (see Section S2 of Data S2). In addition to the correlation having an impact on the cells that are removed, different cost values also affect the way cells are removed. In general, if the cost for subjects receiving the intervention (control) is higher than for the control (intervention) group, assuming equal restart costs, the algorithm tends to remove cells under the intervention (control) condition sooner than those under the control (intervention) condition. If the restart cost for the intervention (control) condition is higher than for the control (intervention), the algorithm avoids generating gaps in the intervention (control) periods during cell removal. If all the costs are the same for both conditions, cells are more likely to be symmetrically removed from both sides. If the per‐participant cost of the control condition is very low and the per‐participant cost of the intervention condition is very high, such that there is extreme imbalance between the participant cost under intervention and control conditions, the optimal design may not resemble the types of staircase designs typically seen in this paper. Instead, these optimal designs might feature an increased number of measurement periods in the control condition, with very few in the intervention condition (see Section S3 of Data S2). We also consider a scenario where there are differential costs to implement the control and intervention conditions in clusters (see Section S4 of Data S2). This example also leads to an optimal design that is distinct from a staircase. Thus, in situations where costs of the intervention and control condition are quite different (at the individual or cluster level), optimal designs may not resemble staircase designs.

We considered a cross‐sectional measurement scheme where a fixed number of participants arrive in each cluster in each period and each participant is measured in only one time period. Other schemes, such as closed‐ and open‐cohort sampling could be accommodated through the inclusion of participant‐level random effects in model (1); we expect results to be similar for such alternative sampling schemes. Furthermore, our methods for seeking cost‐efficient incomplete designs are applicable to all longitudinal cluster randomized trials, including, for example, stepped wedge trials with transition or implementation periods, cluster randomized crossover designs with multiple crossovers, or even already‐incomplete designs such as the staircase. We could also assess the cost efficiency of incomplete designs with unequal cluster‐period sizes, or when linear, rather than categorical, time effects are assumed. Finally, we account for the presence of gaps but not the length of gaps in our cost formula; other functional forms could be used in the formula to capture increasing restart costs associated with longer gaps.

There are numerous potential avenues for further research in this area. This paper focuses on reduced designs that are achieved by eliminating individual sequence‐period cells with the lowest contribution to the cost efficiency metric in an iterative manner starting from a complete stepped wedge design. This is just one way to obtain a series of incomplete designs and evaluate their cost efficiency. Another similar approach was used to iteratively eliminate cluster‐period cells with the lowest information content to obtain incomplete stepped wedge designs to improve the efficiency of a generalized estimating equations approach to the estimation of a treatment effect [36]. Alternative approaches to exploring the space of incomplete stepped wedge designs were discussed in Watson et al. [37]. These papers did not include trial cost considerations directly in their search of the space of incomplete designs, and such integration could be considered.

Further work is required to determine whether trialists and participating clusters would consider designs such as Design 2 in Figures 4 and 6 (i.e., designs where there are some sequences with gaps in data collection) acceptable in practice. These designs require that clusters pause data collection for a length of time, and such pauses may not be suitable in all contexts. We also note that in settings where outcome data is routinely collected such as for a trial embedded into a registry, searching for efficient incomplete designs may not be necessary. However, in situations where data collection is burdensome or recruitment of participants within clusters is likely to be difficult and/or expensive, there will be a need to investigate alternative designs in the manner we have described.

In summary, the work in this paper allows trialists to assess total trial cost together with the power of a design to find cost‐efficient alternatives to a complete stepped wedge design. Incomplete stepped wedge designs with approximately half as many total measurements as the complete design, or in some circumstances an even smaller proportion of measurements, tend to be highly cost‐efficient and may be preferable to the complete design. These incomplete designs reduce both data collection burden and study costs, while maintaining an appropriate level of power. Staircase‐like designs appear to be particularly cost‐efficient variants of the stepped wedge design.

## Conflicts of Interest

The authors declare no conflicts of interest.

## Supporting information


Data S1.

Data S2.

## Data Availability

The project source code and the accompanying app's code are available at https://github.com/EhsanRD/Iterative‐removals‐CE‐diffcl.
