# Supplementary material for: Identifying Less Burdensome and More Cost‐Efficient Incomplete Stepped Wedge Designs for Continuous Outcomes Collected via Repeated Cross‐Sections
Source: Stat Med. 2025 Apr 25;44(8-9):e70067. doi: 10.1002/sim.70067 (PMC12023839; doi:10.1002/sim.70067)
Supplement: Supplementary file 2 — Data S2. [file SIM-44-0-s001.pdf]

Supporting Information
Identifying less burdensome and more cost-efficient incomplete stepped wedge designs for continuous outcomes collected via repeated cross-sections

Ehsan Rezaei-Darzi[1], Jessica Kasza[1], Anisa R Assifi[2], Danielle Mazza[2], Andrew B Forbes[1], and Kelsey L Grantham[1]

[1]School of Public Health and Preventive Medicine, Monash University, Australia
[2]SPHERE NHMRC Centre of Research Excellence, Department of General Practice, School of Public Health and Preventive Medicine, Monash University, Australia

Supplemental Examples

S1| Optimal designs for different ICC-CAC pairs as in the ALLIANCE trial

We consider 9 combinations of ICC and CAC values for the ALLIANCE trial and provide the optimal designs for each. The initial power for all designs is at least 80%. For the ALLIANCE trial example, we assume a 6-period complete stepped wedge design with 7 subjects measured in each cluster-period, with 8 pharmacies allocated to the first and last sequences and 7 pharmacies to the other sequences, a discrete-time decay model with $\rho = 0.05$ and $r = 0.95$, with around 90% power to detect a standardised effect size of 0.26. A restart cost of $g = \$230$ under the intervention group, and $g\prime = \$0$ under the control condition. The cluster recruitment cost $c = \$2,500$, the cost per participant in the intervention condition $p = \$140$, and the cost per participant in the control condition $p\prime = \$80$. The cluster-level cost in the intervention condition $k = \$0$, and in the control condition $k\prime = \$0$.

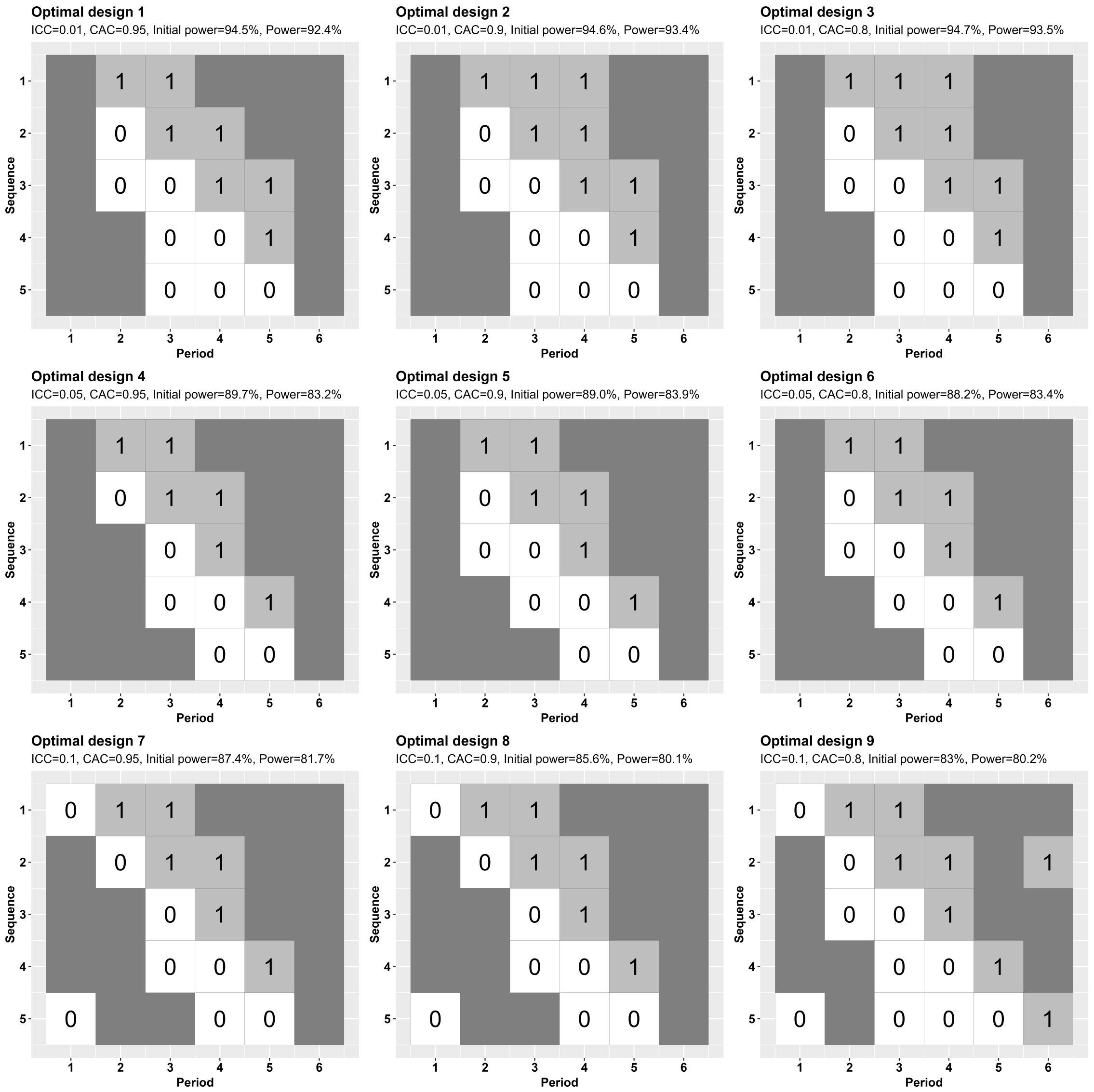

Figure S1: Design schematics of the optimal designs for 9 combinations of ICC and CAC values defined in Section 3.2.

S2| Optimal designs for larger complete stepped wedge designs assuming an exchangeable correlation structure

For both examples here, we assume a 15-period complete stepped wedge design with 50 subjects measured in each cluster-period, one cluster per sequence, an exchangeable model with $\rho = 0.15$ (and $r = 1$), with approximately 90% power to detect a standardized effect size of 0.1. The cluster recruitment cost $c = \$2,500$. For design 1, the restart cost is $g = \$2500$ under the intervention group, and $g\prime = \$0$ under the control condition, with a cost of \$80 per participant under both intervention and control conditions. For design 2, we assume the cost of restarting data collection is \$0 under both conditions and the cost per participant in the intervention condition $p = \$140$, and the cost per participant in the control condition $p\prime = \$80$. The cluster-level cost in the intervention condition $k = \$0$, and in the control condition $k\prime = \$0$.

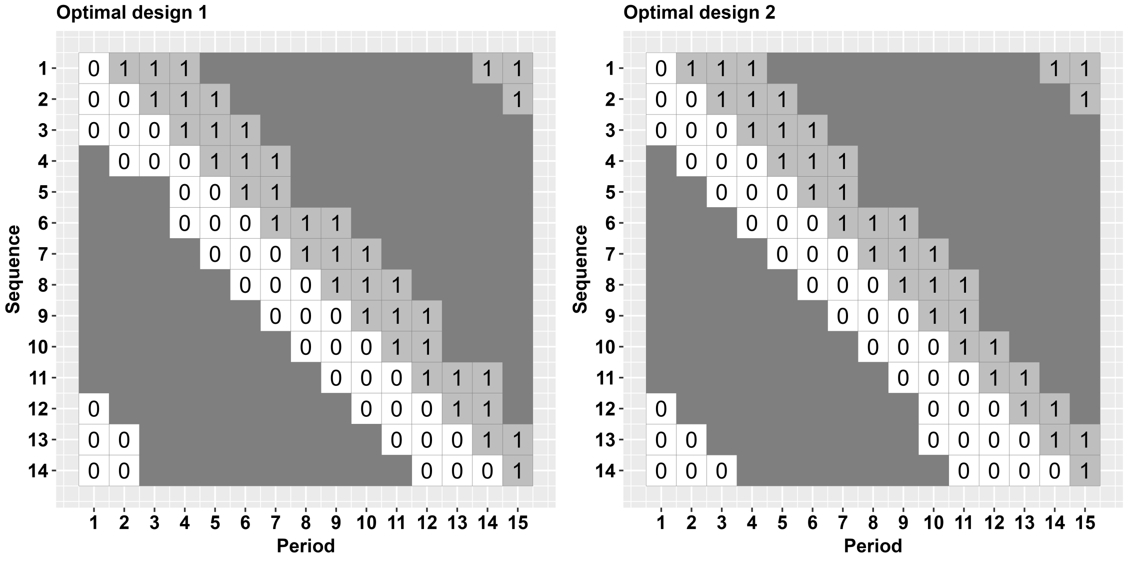

Figure S2: Left: Schematic of the optimal design from Section 3.3, assuming an exchangeable correlation structure instead of a discrete-time decay correlation structure. Right: Schematic of the optimal design from Section 3.4, assuming an exchangeable correlation structure instead of a discrete-time decay correlation structure.

S3| Optimal design for the ALLIANCE trial example with high imbalance between the participant cost under intervention and control conditions

We consider a scenario for the ALLIANCE trial and provide the optimal design for this hypothetical scenario. As in the ALLIANCE trial, we assume a 6-period complete stepped wedge design with 7 subjects measured in each cluster-period, with 8 pharmacies allocated to the first and last sequences and 7 pharmacies to the other sequences, a discrete-time decay model with $\rho = 0.05$ and $r = 0.95$, with around 90% power to detect a standardised effect size of 0.26. A restart cost of $g = \$230$ under the intervention group, and $g\prime = \$0$ under the control condition. The cluster recruitment cost $c = \$2,500$, and now the cost per participant in the intervention condition $p = \$1000$, and the cost per participant in the control condition $p\prime = \$1$. The cluster-level cost in the intervention condition $k = \$0$, and in the control condition $k\prime = \$0$.

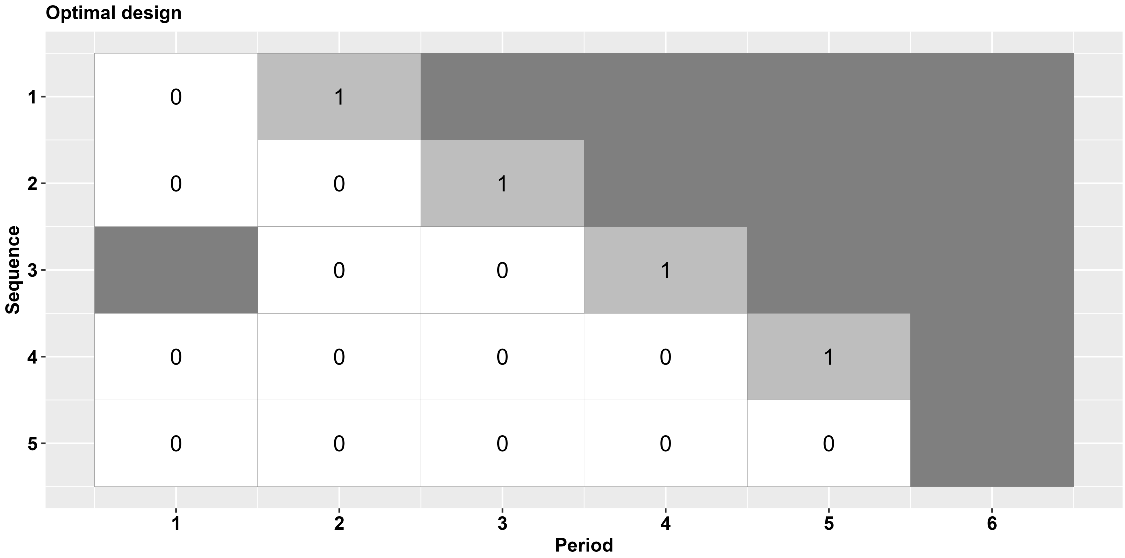

Figure S3: Schematic of the optimal design for the ALLIANCE trial example with an extreme imbalance between the participant cost under intervention and control conditions.

S4| Optimal design for the ALLIANCE trial example with non-zero cluster-level implementation costs for intervention and control

We consider a scenario for the ALLIANCE trial and provide the optimal design for this hypothetical scenario. As in the ALLIANCE trial, we assume a 6-period complete stepped wedge design with 7 subjects measured in each cluster-period, with 8 pharmacies allocated to the first and last sequences and 7 pharmacies to the other sequences, a discrete-time decay model with $\rho = 0.05$ and $r = 0.95$, with around 90% power to detect a standardised effect size of 0.26. A restart cost of $g = \$230$ under the intervention group, and $g\prime = \$0$ under the control condition. The cluster recruitment cost $c = \$2,500$, the cost per participant in the intervention condition $p = \$140$, and the cost per participant in the control condition $p\prime = \$0$. We assume that the cluster-level cost in the intervention condition $k = \$1000$, and in the control condition $k\prime = \$0$.

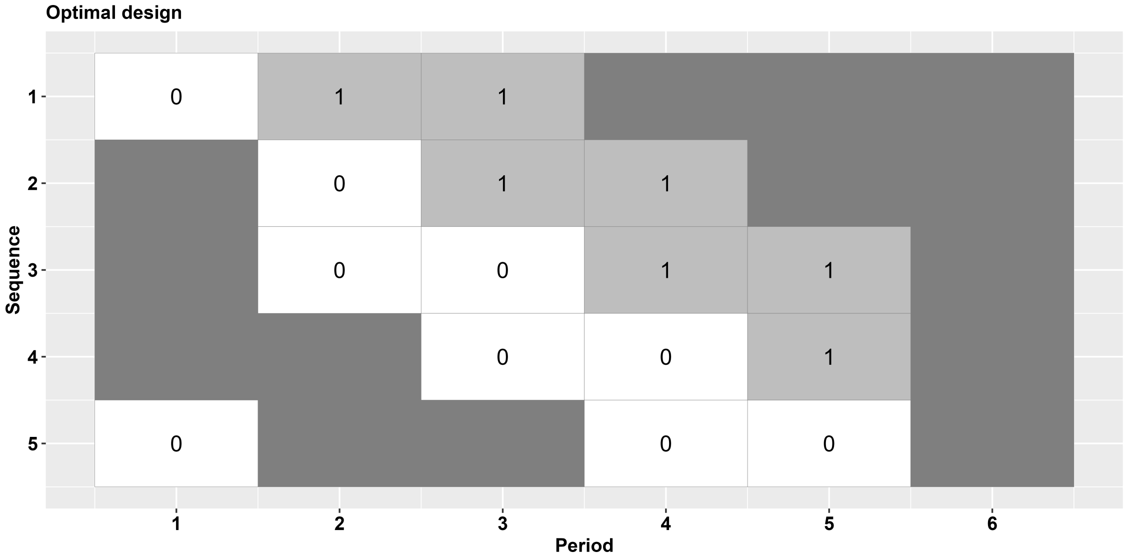

Figure S4: Schematic of the optimal design for the ALLIANCE trial example with an extreme imbalance between the cluster-level cost under intervention and control conditions.
